# Chagas Disease in Spain: Need for Further Public Health Measures

**DOI:** 10.1371/journal.pntd.0001962

**Published:** 2012-12-27

**Authors:** Miriam Navarro, Bárbara Navaza, Anne Guionnet, Rogelio López-Vélez

**Affiliations:** Tropical Medicine and Clinical Parasitology, Infectious Diseases Department, Hospital Universitario Ramón y Cajal, Madrid, Spain; Institute of Tropical Medicine, Belgium

## Introduction

Chagas disease, caused by the protozoa *Trypanosoma cruzi*, is endemic from Latin America and is considered a neglected tropical disease (NTD). An estimated 8 million people worldwide are infected, the country with the highest disease burden being Bolivia. Chagas disease has overcome borders, and it is no longer restricted to endemic countries. Nowadays, it can be found in the Unites States, Canada, Europe, Japan, and Australia, mainly due to mobility of population. Spain ranks second only to the United States in the list of countries receiving migrants from Latin America, and it is the European country with the highest prevalence of Chagas disease, therefore posing a challenge in terms of public health [1].

*T. cruzi* transmission is feasible in vector-free world regions. The main nonvectorial routes are congenital transmission, blood transfusion, and solid organ transplant. In fact, in Europe as well as in other nonendemic areas, there have been several cases of vertical transmission of *T. cruzi* and also transmission through infected tissue or blood products. Many nonendemic countries have not yet established official guidelines to avoid these routes [1], [2]. Mandatory screening of blood donors at risk for *T. cruzi* infection has been implemented since October 2005 in Spain targeting donors born in endemic areas, those whose mothers were born in endemic areas, and people who received blood transfusions in endemic areas [3]. Although not included in the Royal Decree, many blood banks also screen individuals who have resided in endemic areas for more than 2 months. In Spain, there is a national law that regulates the activity of tissue banks; moreover, the Spanish Society of Tropical Medicine and International Health has published a document in order to establish the guidelines to be followed in case a potential donor or a tissue or organ recipient could be affected by Chagas disease [4].

According to the recommendations of the WHO experts on the control and management of congenital Chagas disease, screening should be carried out during pregnancy to detect mothers who carry the infection and are at risk of transmitting the infection to their offspring. Furthermore, the WHO states that cases of congenital *T. cruzi* infection should be treated as soon as the diagnosis is confirmed using benznidazole or nifurtimox [5]. In Spain, systematic detection of congenital infection is not performed at the national level [1]; only two regions (Autonomous Community of Valencia and Catalonia) have a specific protocol. Currently, several scientific groups have drafted a clinical guide, which will be published shortly, about diagnosis, follow-up, and treatment of pregnant women and children with Chagas disease.

Regarding treatment of infected newborns, the WHO recommendations are followed [5], using benznidazole as the first-line treatment option for Chagas disease in children and adults in Spain. Stocks of this drug ran out at the end of last year, which will result in Chagas disease becoming a NTD also in developed countries [6]. Until March 2012, this drug was only produced by the Brazilian state-owned laboratory LAFEPE. At that moment, the drug started to be produced also in Argentina through a private-public partnership led by Fundación Mundo Sano. Benznidazole is available in Spain for all patients from the second half of November 2012.

Estimating the burden of Chagas disease in nonendemic countries is crucial in order to plan preventive measures and to determine the resources for screening and treatment. Estimates are normally calculated according to the number of Latin Americans registered in each country and to the infection rates in their countries of origin. Thus, it is assumed that the prevalence of the infection in the host country is the same as that in the country of origin, this being the main limitation for achieving accurate estimations.

In the past recent years, estimates on the expected number of migrants with *T. cruzi* infection have been carried out in Spain. The last one yields a figure of 48,000–86,000 cases, based on the Bolivians' infection rates of three studies performed only in two regions of Spain [7]. Being aware of the heterogeneous distribution of immigration, studies performed in different geographical regions of Spain were taken into account in this article in order to reach a comprehensive approach.

## In Order to Get an Accurate Estimate of the Prevalence of Chagas Disease in Spain, Several Factors Were Reconsidered

• Current number of registered migrants from Latin America living in Spain, paying special attention to those coming from Bolivia as they represent the population with the highest disease burden. It is important to work with updated statistics on migrations—for example, more than 22,000 (4.3%) Bolivians left Spain during 2009 [8]. In fact, the number of Bolivian migrants living in Spain in 2009 rose up to 230,700 [9]. Currently, there are 1,569,355 registered migrants from Latin America living in Spain, out of which 199,080 (12.7%) come from Bolivia [10].

*• T. cruzi* infection rates in adult Bolivian migrants (aged over 17) carried out in health centers other than Spanish specialized clinics such as centers for imported diseases, Tropical Medicine Centers, or International Health Centers. The reason is that in specialized services, the prevalence may be overestimated (over 31% [11]), as many patients are referred by other centers where the disease has already been diagnosed. Locations chosen for this purpose were nonclinical settings (migrants' associations and nongovernmental organizations focused on Latin American migrants), blood donation centers, and maternity hospitals.

Seven studies carried out between 2005 and 2010 (2010 being the final year for which data are considered) were recorded (Table 1) [12]–[18]. Although other studies were performed in similar settings, they were not taken into consideration because diagnosis was not performed using two serological tests (according to WHO guidelines): it was performed with an immunochromatographic (IC) test, and only the positive and doubtful results were confirmed. Those IC tests were not sufficiently sensitive for them to be recommended as screening tools [16], [19].

**Table 1:** Seroprevalence of *T. cruzi* infection among adult migrants (aged over 17) in Spain (2005–2010).

Author, Year of Publication	Setting/Region	Global *T. cruzi* Prevalence % (Number/Total)	*T. cruzi* Prevalence Among Bolivians % (Number/Total)	Percentage of Bolivians Among Infected Patients % (Number/Total)
Barona-Vilar C, 2011 [18]	Maternity hospitals/Valencia	11.4 (226/1,975)	34.1 (214/628)	94.7 (214/226)
Flores-Chávez M, 2011 [17]	Maternity hospitals/Madrid	3.9 (152/3,839)	11.4 (91/798)	59.9 (91/152)
Navarro M, 2011* [16]	Non-clinical/Madrid, Cádiz, Alicante	16.4 (44/268)	21.7 (44/203)	100 (44/44)
Lucas RM, 2009 [15]	Maternity hospital/Valencia	9.7 (37/383)	26.0 (20/77)	54.1 (20/37)
Muñoz J, 2009 [14]	Maternity hospital/Catalonia	3.4 (46/1,350)	22.2 (42/189)	91.3 (42/46)
Paricio-Talayero JM, 2008 [13]	Maternity hospitals/Valencia, Alicante	4.8 (29/624)	17.5 (24/137)	82.8 (24/29)
Pirón M, 2008 [12]	Blood bank/Catalonia	0.7 (10/1,524)	10.2 (6/59)	60 (6/10)
Total % (95% CI)		5.5 (5.0–5.9)	21.1 (19.3–22.9)	81.1 (77.7–84.5)

* Extracted from: Navarro M, et al. Eurosurveillance 2011. Data calculated by age and sex.

Five out of the seven studies recorded were performed in women (maternity hospitals). The other two had female and male participants. Data coming from the study carried out at a blood bank are not disaggregated by sex. Data from the other study [16] were extracted and calculated separately by age and sex. It showed no significant differences between seroprevalence in women (23.1%, 95% CI 15.5–30.7) and men (19.2%, 95% CI 9.5–28.9), these rates being similar to the global one (21.7%, 95% CI 15.8–27.6) (Table 1, [11]). Therefore, no data divided by sex are presented.

## Prevalence of *T. cruzi* Infection in Spain

• There are 162,095 Bolivian adults (older than 19 years old) living in Spain [10]. The distribution of Bolivian adults regarding sex is 97,215 women (60%) and 64,880 men.

• Applying an average rate of 21.1% (calculated from the averages of the seven collected studies) to the Bolivian adult immigrants (both male and female), an estimate of potentially infected immigrants from Bolivia was obtained, yielding a figure of 34,202. Taking into account that around 81% of Chagas disease cases in Spain occur among Bolivian patients (average percentage of the seven studies; Table 1), 42,173 total estimated cases among adult immigrants from endemic areas were observed.

Heterogeneity in seroprevalences can be mostly explained by the diversity of migrants screened in the different studies. In some studies, most of the patients were from Bolivia and, therefore, *T. cruzi* prevalence was high: those studies with preventive programs focused on Bolivians [16], and those performed in cities such as Valencia where the proportion of Bolivians is very high [18]. In other studies where the country of origin of the screened migrants was more diverse, including for example patients from Ecuador and Colombia, the prevalence was not so high [12]–[15], [17]. The specific area of origin of screened Bolivians was only mentioned in one study [16], where the majority came from hiperendemic areas. Nonetheless, in cities such as Valencia or Barcelona [14], [18], the Bolivian population comes mainly from hiperendemic areas like Santa Cruz or Cochabamba, and there were less cases of patients coming from less endemic areas like La Paz.

With an average proportion of 25% of cases suffering from clinical disease (around 18% cardiac, 8% digestive, and 2% cardio-digestive involvement [11], [20], [21]), this calculation results in a total of 10,543 adult individuals who are likely to have Chagas disease with cardiac and/or digestive involvement.

**Figure 1:**
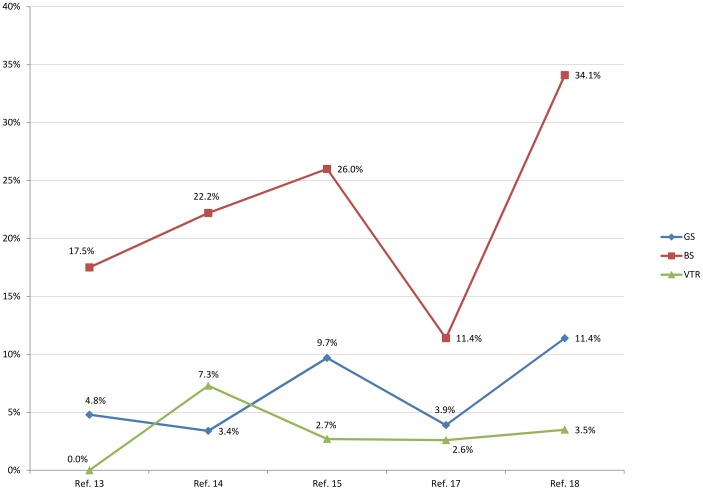
*T. cruzi* seroprevalence and vertical transmission rate in the five studies performed in maternity hospitals. GS, Global *T. cruzi* seroprevalence; BS, *T. cruzi* seroprevalence among Bolivians; VTR, vertical transmission rate; Ref, reference.

• Currently, 93,557 Bolivian women of childbearing age (15–49 years old) are living in Spain [10]. Applying the seroprevalence of maternity hospitals (21.4%) and considering that around 80% of cases occur among Bolivian women, the estimated number of cases among women of childbearing age was 25,026. Average vertical transmission rate of the five selected studies that were performed in maternity hospitals was 3.3%. (Figure 1).

## Conclusion

Within this article we highlight that:

- This is a more accurate estimate of the *T. cruzi* infection rate because it is based on migrants' seroprevalences and not on those found in their countries of origin, providing a current picture of what Chagas disease burden represents in nonendemic countries, focusing specially in Spain, the country with the highest prevalence outside the Americas.
- This figure does not consider the approximately 10,000 migrants that were born in Bolivia but already have Spanish citizenship [22], nor those undocumented migrants, and neither the pediatric cases [7]. Thus, the actual number could be even higher.
- These figures, if certain, point out that only about 10% of the cases have been already diagnosed. In the context of a silent disease like Chagas, this poses a great challenge for nonendemic countries.
- This situation requires public health actions such as targeted screening programs in order to get an early diagnosis and treatment for patients and to avoid nonvectorial transmission. In Spain, although screening for *T. cruzi* is currently performed for at-risk blood and solid organ donors and it is recommended for gestating women coming from endemic areas, there are no official national guidelines for screening pregnant women.

Early detection and treatment of neonates soon after delivery result in the best therapeutic results and may prevent the establishment of the disease and the development of future complications in adulthood. Studies performed in Bolivia and Spain showed that screening pregnant women from endemic areas and their newborns is a better option than no screening in terms of costs [23] and quality of life for patients [23], [24].

Therefore, screening programs should be mandatory among gestating women (and highly recommended among young women) from endemic areas: women of childbearing age represent more than 60% of the overall estimated cases, the benefits of early treatment in infected newborns are widely demonstrated, and the specific treatment before pregnancy would decline mother-to-child transmission rates.
